# Resolution of hypokalemia following nephrectomy for a presumed renal cell carcinoma revealing a large angiomyolipoma: A case report

**DOI:** 10.1016/j.eucr.2025.103108

**Published:** 2025-07-01

**Authors:** Fatemeh Khajeh, Shahryar Zeighami, Ali Ariafar, Zahra Bazargani, Hossein Hakimelahi

**Affiliations:** a Pathology Department, Fasa University of Medical Sciences, Urology Department, Iran; b Urology Department, Shiraz University of Medical Sciences, Iran; c Cancer Research Center of Shiraz Medical University, Iran; d Clinical Research Development Unit, Department of Pediatrics, Valiasr Hospital, Fasa University of Medical Sciences, Fasa, Iran

**Keywords:** Renal angiomyolipoma, Renal cell carcinoma, Hypokalemia, Nephrectomy, Paraneoplastic syndrome

## Abstract

Renal angiomyolipomas (AMLs) are benign tumors that can mimic renal cell carcinoma (RCC) when fat content is minimal. We report a 56-year-old male with recurrent paralysis and hypokalemia, presumed to be a paraneoplastic syndrome of RCC, prompting radical nephrectomy. Histopathology and immunohistochemistry (IHC) revealed an 8 cm AML, and hypokalemia resolved post-surgery. This case underscores the diagnostic challenge of minimal-fat AMLs and suggests that an accurate preoperative diagnosis could have enabled kidney-sparing surgery.

## 1 Introduction

Renal angiomyolipomas (AMLs) are benign neoplasms composed of fat, smooth muscle, and vascular elements, frequently associated with tuberous sclerosis complex (TSC) or lymphangioleiomyomatosis (LAM). Minimal-fat AMLs are difficult to distinguish from RCC on imaging, often leading to misdiagnosis and overtreatment. Hypokalemia, typically linked to RCC as a paraneoplastic syndrome, is rarely reported with AMLs, with only a few documented cases in the literature. We present a case where hypokalemia and a presumed RCC diagnosis led to nephrectomy, with an unexpected AML finding confirmed by IHC, raising questions about optimal management.

## 2 Case presentation

A 56-year-old male presented with recurrent episodes of severe lower extremity weakness, paralysis, and fatigue, necessitating three hospital admissions. His history and physical examination were unremarkable, with no signs of TSC or LAM. Laboratory findings revealed hypokalemia (potassium 2.8 mEq/L; reference 3.5–5 mEq/L), microscopic hematuria (5–8 RBCs/10 HPF), and normal creatinine (1.1 mg/dL), sodium (138 mEq/L), hemoglobin (13.8 g/dL), and hematocrit (41 %). Urine pH was 6.2. Nerve conduction velocity (NCV) and electromyography (EMG) were inconclusive.

Hypokalemia was managed with potassium chloride (KCl) infusion (60 mEq in 1000 cc saline over 10 hours). Persistent hematuria and abdominal distention prompted abdominal ultrasonography, identifying an 8 × 6 cm mass in the upper pole of the left kidney. A CT scan confirmed a solid 8 × 6 cm mass without invasion or metastasis, interpreted as RCC. Adrenal glands were normal. Given the size, hematuria, and hypokalemia, suspected as a paraneoplastic syndrome of RCC, radical left nephrectomy was performed.

Pathology revealed a well-defined, creamy-brown, rubbery mass (8 × 7 × 6 cm) with a biphasic pattern. Immunohistochemistry (IHC) confirmed AML with positive staining for HMB-45 and Melan-A, markers of perivascular epithelioid cell differentiation, and negative staining for CK7 and PAX8, ruling out RCC. Post-operatively, potassium normalized (4.3 mEq/L) within 48 hours without supplementation, and weakness resolved. At one-year follow-up, the patient remained well with stable renal function.

## 3 Discussion

The preoperative diagnosis of RCC in this case was based on the mass's large size, minimal fat content on imaging, and hypokalemia, presumed to be a paraneoplastic syndrome linked to RCC (e.g., ectopic renin or cytokine production)^1^. Minimal-fat AMLs are notoriously difficult to differentiate from RCC on imaging, as CT and MRI lack specificity in these cases^2^. IHC was pivotal: positive HMB-45 and Melan-A staining confirmed AML, reflecting its mesenchymal origin, while negative CK7 and PAX8 excluded RCC, which typically expresses these epithelial markers in clear cell and papillary subtypes^3^. Had AML been suspected preoperatively, tumor enucleation or partial nephrectomy could have preserved renal parenchyma, avoiding radical nephrectomy—a critical consideration given AML's benign nature^4^. The resolution of hypokalemia post-surgery suggests a tumor-related mechanism, possibly mechanical tubular disruption or a paraneoplastic effect, though the latter is less documented with AMLs than RCC.^1^ This case underscores the diagnostic pitfalls of minimal-fat AMLs and the potential for misdirected aggressive surgery when RCC is presumed, highlighting the diagnostic precision of IHC with HMB-45, Melan-A, CK7, and PAX8.

## 4 Conclusion

This case illustrates a rare AML presenting with hypokalemia, initially misdiagnosed as RCC, leading to nephrectomy rather than kidney-sparing surgery. IHC confirmation with positive HMB-45 and Melan-A, and negative CK7 and PAX8, was critical in distinguishing AML from RCC. Accurate preoperative differentiation of minimal-fat AMLs from RCC remains challenging but is crucial to optimize management. The resolution of hypokalemia post-resection suggests a tumor-related etiology, warranting further investigation into AML-associated paraneoplastic phenomena.

Ethics Approval: As no direct patient interaction occurred, institutional guidelines deemed formal ethics approval unnecessary.

## CRediT authorship contribution statement

**Fatemeh Khajeh:** Writing – review & editing, Writing – original draft, Validation, Formal analysis, Data curation, Conceptualization. **Shahryar Zeighami:** Validation, Supervision, Project administration, Data curation, Conceptualization. **Ali Ariafar:** Validation, Supervision, Data curation. **Zahra Bazargani:** Visualization, Validation, Supervision. **Hossein Hakimelahi:** Visualization, Writing – review & editing.

## Data availability

The data supporting this case report are available in our clinic and pathology lab files.

## Funding

No funding was received for this case report from any public, commercial, or not-for-profit entities.

## Declaration of competing interest

The authors declare no financial or personal conflicts of interest.
